# EF24 Suppresses Cholangiocellular Carcinoma Progression, Inhibits STAT3 Phosphorylation, and Induces Apoptosis via ROS-Mediated Oxidative Stress

**DOI:** 10.1155/2019/8701824

**Published:** 2019-03-04

**Authors:** Savita Bisht, Jens Nolting, Jörg Wenzel, Peter Brossart, Georg Feldmann

**Affiliations:** ^1^Department of Internal Medicine 3, Center of Integrated Oncology (CIO) Cologne-Bonn, University Hospital of Bonn, Germany; ^2^Department of Dermatology and Allergy, Center of Integrated Oncology (CIO) Cologne-Bonn, University Hospital of Bonn, Germany

## Abstract

Therapeutic options for advanced stage cholangiocellular carcinoma (CCC) are very limited as of today and patients carry an exceptionally poor overall prognosis. In recent years, increasing evidence has been accumulated to suggest that malignant cells widely show increased intrinsic ROS levels and exhibit altered redox profiles as compared to normal counterparts, opening up potential avenues for therapeutic intervention. This study provides preclinical experimental evidence of therapeutic activity of the curcumin analog EF24 in cholangiocarcinoma models. In CCC cell lines, EF24 inhibited cell viability and induced apoptosis through excessive ROS generation. Moreover, administration of EF24 led to depletion of total intracellular GSH levels, induced mitochondrial depolarization, and abrogated STAT3 phosphorylation. Of interest, these effects were readily averted by treating the cells with exogenous antioxidants such as N-acetyl cysteine (NAC) or glutathione monoethyl ester (GEE). In vivo, EF24, solubilized using a cyclodextrin formulation, significantly suppressed the growth of tumor xenografts without exhibiting any toxic adverse effects. Immunohistochemical analysis of extracted tumor tissues demonstrated reduced nuclear staining for Ki-67 and downregulation of phospho-STAT3 as well as strong staining for oxidative stress biomarker 8-OHdG. Therefore, the data presented here suggest EF24 as potential therapeutic compound against CCC which might act at least to some extent through ROS-induced oxidative damage, subsequently inducing apoptosis. Further evaluation of this approach should be carried out in future follow-up studies.

## 1. Introduction

Cholangiocellular carcinoma (CCC) is the second most common cancer of the hepatobiliary tract and arises from malignant transformation of cholangiocytes lining intra- and extrahepatic bile ducts [1, 2]. Although cholangiocarcinomas comprise 10-15% of all hepatobiliary neoplasms, their incidence and mortality rates are rising worldwide [3]. Most of the currently available treatment options for advanced CCC such as biliary drainage, systemic therapy with tyrosine kinase inhibitors, conventional chemotherapy, or photodynamic therapy show limited effects on overall survival and quality of life. Thus, novel therapeutic strategies are needed to improve quality of life and outcome for cholangiocarcinoma patients.

In recent years, ROS stress in cancer cells and its potential therapeutic implications have emerged as a promising area of research. Mounting evidences suggest that, due to increased metabolic activity, oncogenic stimulation, and mitochondrial malfunction, cancerous cells produce higher levels of ROS and are thus constantly in a state of increased chronic oxidative stress as compared to noncancerous counterparts [4–8]. Although moderate increase in ROS production promotes carcinogenesis and cancer progression due to their stimulating effects on cell growth and proliferation [9–14], excessive production and accumulation of ROS can cause irreversible cellular damage triggering cell death [15]. Of interest, increased basal oxidative stress in cancerous and transformed cells renders them highly dependent on antioxidant systems to counteract the damaging effects of ROS [16–19], opening up potential avenues for therapeutic intervention by agents that confer further oxidative pressure.

This present study identifies the curcumin analog EF24 as potential therapeutic agent with the potential to target cholangiocellular carcinoma cells by enhancing excessive ROS production while at the same time quenching antioxidative response.

## 2. Materials and Methods

### 2.1. Reagents

Curcumin and its analog EF24 were purchased from Sigma-Aldrich (Steinheim, Germany). Stock solutions of each (20 mM) were prepared in DMSO and stored in aliquots at -20°C. The compounds were diluted in culture media prior to each experiment. N-acetyl-L-cysteine (NAC) was also obtained from Sigma-Aldrich and dissolved in double distilled water. Recombinant human IL-6 (RELIATech GmbH), Annexin V/PI kit (BD Biosciences, Schwerte, Germany), Total ROS/Superoxide Detection Kit (Enzo Life Sciences GmbH, Lörrach, Germany), and ApoGSH™ Glutathione Colorimetric Assay Kit (BioVision, Milpitas, CA) were used according to the respective protocol recommended by the manufacturer.

### 2.2. Cell Culture

Human cholangiocarcinoma cell lines were grown in RPMI media (PAA Laboratories, Pasching, Austria) supplemented with 10% fetal bovine serum, 1x penicillin-streptomycin (PAA Laboratories, Pasching, Austria), and 5 *μ*g/mL of plasmocin (InvivoGen, San Diego, CA). All cell lines were grown in a humidified atmosphere at 37°C in the presence of 5% CO_2_ and were routinely tested for mycoplasma infection using a PCR-based assay as described elsewhere [20]. Cells were treated with EF24 dissolved in DMSO with final concentrations of DMSO not exceeding 0.1%. DMSO-treated cells were used as mock controls in all the experiments.

SNU478 is a human biliary tract cancer cell line derived from a histologically confirmed Ampulla of Vater carcinoma in a Korean patient; its parent tumor was found to represent a poorly differentiated adenocarcinoma with signet ring cell feature and infiltrated to the pancreas along the interstitial space as a single cell or cell cords [21]. HuCC-T1 is one of the most widely used human cholangiocellular carcinoma cell lines, originally established in vitro from the malignant cells of ascites of a 56-year-old patient and proven to faithfully preserve key genetic alterations and protein expression patterns reminiscent of human biliary tract cancer [22].

### 2.3. Cell Viability (MTS) Assays

Cell viability of cholangiocarcinoma cell lines was determined using MTS assays as described previously [23, 24]. Briefly, 5000 cells/well were plated in 96-well plates and were treated with increasing concentrations of 0, 0.1, 0.5, 1.0, and 5.0 *μ*M of free curcumin or equivalent doses of EF24, respectively, for 24, 48, and 72 hours, at which point the assay was terminated, and relative growth inhibition compared to mock-treated cells was measured using CellTiter 96 reagent, as recommended in the manufacturer's protocol. All experiments were set up in triplicates to determine means and standard deviations.

### 2.4. Clonogenicity Assays

Cells were seeded at a density of 1,000 per well into six well plates and treated with increasing concentrations of EF24 for 24 hours. DMSO was added in control wells. On the following day, media-containing drug was removed and the cells were washed with PBS. Fresh growth medium was then added to the cells. Once colonies became visible, cells were fixed with 70% ethanol and stained with 0.05% (w/v) crystal violet solution (Sigma-Aldrich, Steinheim, Germany).

### 2.5. Annexin V-FITC/PI Apoptosis Assays

SNU478 and HuCC-T1 cells were treated with 1 *μ*M or 5 *μ*M of EF24 for 24 hours. DMSO was used as mock control. After treatment, cells were harvested, washed in ice-cold PBS, and resuspended in 500 *μ*L of 1x binding buffer containing 5 *μ*l of Annexin-V and 5 *μ*L of PI solution. Cells were incubated at room temperature for 15-20 minutes in the dark and were then immediately analyzed using a BD FACSCanto II flow cytometer (BD Biosciences, San Jose, CA, USA).

### 2.6. Measurement of Intracellular Reactive Oxygen Species (ROS)

Intracellular ROS and superoxide levels were measured using Total ROS/Superoxide Detection Kit (Enzo Life Sciences, Lörrach, Germany). Briefly, cells were cultured and treated with appropriate concentration of EF24 in the absence and presence of NAC. After 8 hours, media was removed and the cells were washed twice with 1x wash buffer. The cells were then trypsinized, centrifuged for 5 min at 400 x g, and further resuspended in 500 *μ*l of ROS/superoxide detection solution. Cells were stained at 37°C for 30 min in the dark and immediately analyzed by flow cytometry.

### 2.7. Measurement of Total Cellular Glutathione (GSH) Content

Total glutathione (GSH) levels in cell extracts were measured using Total Glutathione Colorimetric Assay Kit (BioVision). Briefly, cells treated with DMSO or EF24 were lysed in ice-cold glutathione buffer and deproteinized using ice-cold 5% (w/v) sulfosalicylic acid (SSA). The lysates were centrifuged at 8000 x g for 10 minutes, and the collected supernatants were used for glutathione assays according to the manufacturer's protocol. The amount of GSH was calculated from a standard curve generated under similar conditions.

### 2.8. Mitochondrial Membrane Potential Measurement (Δ*ψ*m)

One million of cells untreated or treated with different concentrations of EF24 were resuspended in 1 mL of RPMI medium and loaded with 250 nM fluorescent probe, tetramethyl rhodamine methyl ester (TMRM). Cells were then incubated for 25 minutes at 37°C in the dark, washed twice with PBS, and resuspended again in FACS buffer prior to data acquisition by flow cytometry.

### 2.9. Western Blots

SNU478 and HuCC-T1 cells were treated with two different concentrations of EF24 (1 *μ*M or 5 *μ*M) or DMSO for 24 h. Next, cells were lysed using radioimmunoprecipitation assay buffer (RIPA: 1% Igepal CA 630, 0.5% sodium deoxycholate, 0.1% SDS, and 2 mM EDTA) supplemented with protease and phosphatase inhibitor cocktails (Sigma-Aldrich, Steinheim, Germany). 50 *μ*g of total protein was separated using 4-12% NuPAGE Bis-Tris Gels (Life Technologies, Darmstadt, Germany) and transferred onto PVDF membranes (Millipore, Billerica, MA, USA). The blots were blocked using either 5% (w/v) BSA or 5% (w/v) milk in TBST for 1 hour and then probed using primary antibodies against BAX, BCL2, phospho-STAT3, STAT3, caspase 3, cleaved caspase 3, PARP, or GAPDH (1:1000, Cell signaling, Danvers, MA). HRP-coupled secondary antibodies directed against rabbit or mouse IgG, respectively (1:2000. Cell Signaling, Danvers, MA), were subsequently used. Detection was performed as previously described [25]. Quantification was done by densitometry using ImageJ analysis software (https://imagej.nih.gov/ij/).

### 2.10. Xenograft Studies

All animal experiments described were approved by the local authorities. Mice were maintained and handled in accordance with the guidelines of the Federation of European Laboratory Animal Science Associations (FELASA). SNU478 xenografts were generated by injecting 2.5 x 10^6^ cells resuspended in a total volume of 200 *μ*L [PBS/Matrigel (BD Biosciences), 1:1 (v/v), prechilled to 4°C] subcutaneously into the flanks of 6-8-week-old NOD-SCID HPRT mice [26]. After two weeks, subcutaneous tumors were measured using digital calipers (Milomex, Pulloxhill, UK) and tumor volumes V were calculated as V=1/2(ab^2^), where a is the longest and b is the shortest orthogonal tumor diameter [27]. Mice were then randomized into two cohorts (n=6 animals per cohort) and assigned to either of the following treatment regimens: (i) 2-hydroxypropyl-beta-cyclodextrin as vehicle only or (ii) EF24 solubilized in 10% (w/v) 2-hydroxypropyl-beta-cyclodextrin (AppliChem, Darmstadt, Germany) at a daily dose of 10 mg/kg i.p. Tumor volumes and body weights were measured weekly. After three weeks, tumors were harvested and fixed in 10% buffered formalin solution for further immunohistochemical analysis.

### 2.11. Statistical Analysis

Two-tailed Student's* t*-test and Mann-Whitney* U* test were performed using GraphPad Prism for Windows version 6. Kruskal-Wallis analyses were done using SPSS for Microsoft Windows. p<0.05 was regarded as statistically significant. Unless indicated otherwise, results are shown as mean ± standard deviation.

## 3. Results

### 3.1. EF24 Inhibits Growth and Clonogenicity of Cholangiocarcinoma Cells

**Figure 1:**
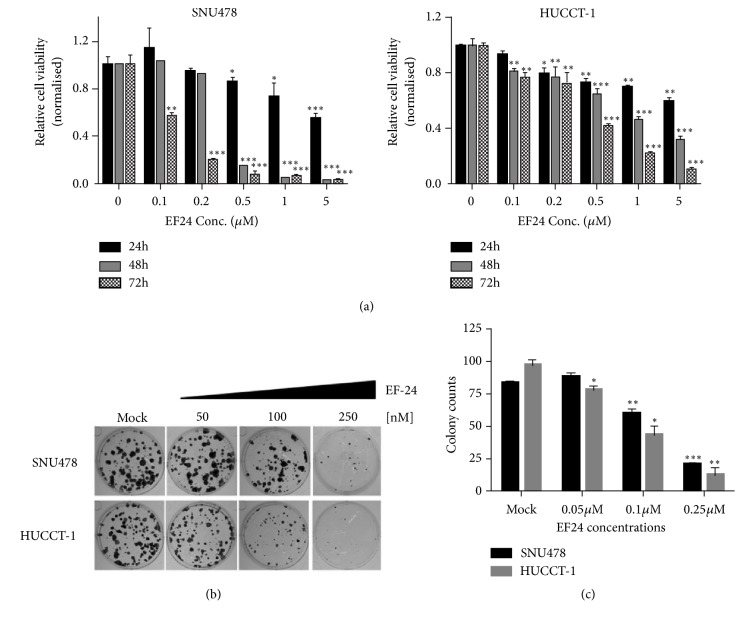
*EF24 treatment inhibits growth and clonogenic potential of cholangiocarcinoma cells.* (a) Treatment of SNU478 and HuCC-T1 cells with EF24 resulted in significantly reduced net cell growth as assessed by cell viability (MTS) assays, in a dose- and time-dependent manner. EF24 significantly reduced the number as well as average size of colonies formed in replating efficiency assays. Representative images (b) and colony counts (c) of three independent experiments are shown (“*∗*” indicates p<0.05, “*∗∗*” indicates p<0.01, and “*∗∗∗*” indicates p<0.001 as compared to mock treated controls, resp.).

Net cell growth of established cholangiocarcinoma cell lines SNU478 and HuCC-T1 was evaluated in the presence of increasing concentrations of EF24 or its parent compound curcumin, respectively, by means of cell viability (MTS) assays. As shown in Figure 1(a), EF24 potently inhibited net cell growth of both SNU478 or HuCC-T1 cells, respectively, in a dose- and time-dependent manner. Next, the in vitro efficacy of EF24 on clonogenicity of SNU478 and HuCC-T1 cells was determined using single cell suspension replating assays (Figure 1(b)). Here, EF24 drastically reduced the ability of both cell lines to form colonies at concentrations in the range of 100-250 nM (Figure 1(c)). As opposed to this observation, the parent compound curcumin did not cause any tangible growth inhibition at concentrations of up to 5 *μ*M in MTS assays and also did not show any effect on the clonogenic potential of SNU478 or HuCC-T1 cells at concentrations of up to 1 *μ*M (Supplementary Figure 1).

### 3.2. EF24 Induces Apoptosis in CCC Cells

**Figure 2:**
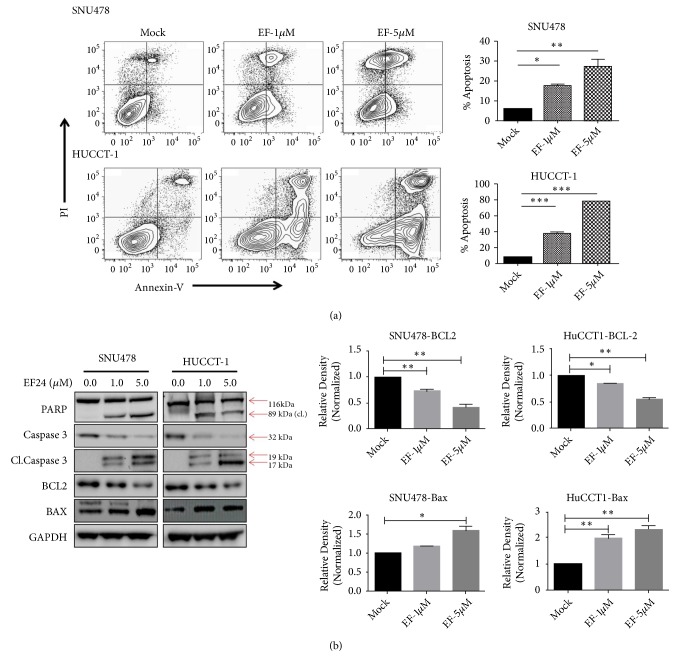
*EF24 induces apoptosis in cholangiocarcinoma cells in vitro.* EF24 significantly induced apoptosis in SNU478 and HuCC-T1 cell lines determined by flow cytometry analysis of Annexin V positive cells (a) as well as by Western blot analysis (b). The bar diagrams show quantification of Western blot results; “*∗*” stands for p<0.05; “*∗∗*” stands for p<0.01 as compared to mock-treated control.

In order to better understand the mechanism underlying the observed reduction of in vitro net CCC cell growth caused by treatment with EF24, we next evaluated its effect on apoptosis using Annexin binding assays. Flow cytometry analysis of Annexin-V/PI stained SNU478 and HuCC-T1 cells revealed significant induction of apoptosis, including an increase in both early and late apoptotic states, upon treatment with EF24 in a concentration-dependent manner (Figure 2(a)). In line with this finding, after treatment with EF24 (at concentrations of 1 *μ*M or 5 *μ*M, resp.) both SNU478 and HuCC-T1 cells showed increased levels of cleaved PARP and cleaved caspase 3 as biomarkers of apoptosis, as well as reduction in BCL2 (antiapoptotic) and concomitant increase in BAX (proapoptotic) protein levels as determined using Western blot analysis (Figure 2(b)).

### 3.3. Increase in Oxidative Stress Plays an Essential Role in EF24-Induced Apoptosis and Cell Death

**Figure 3:**
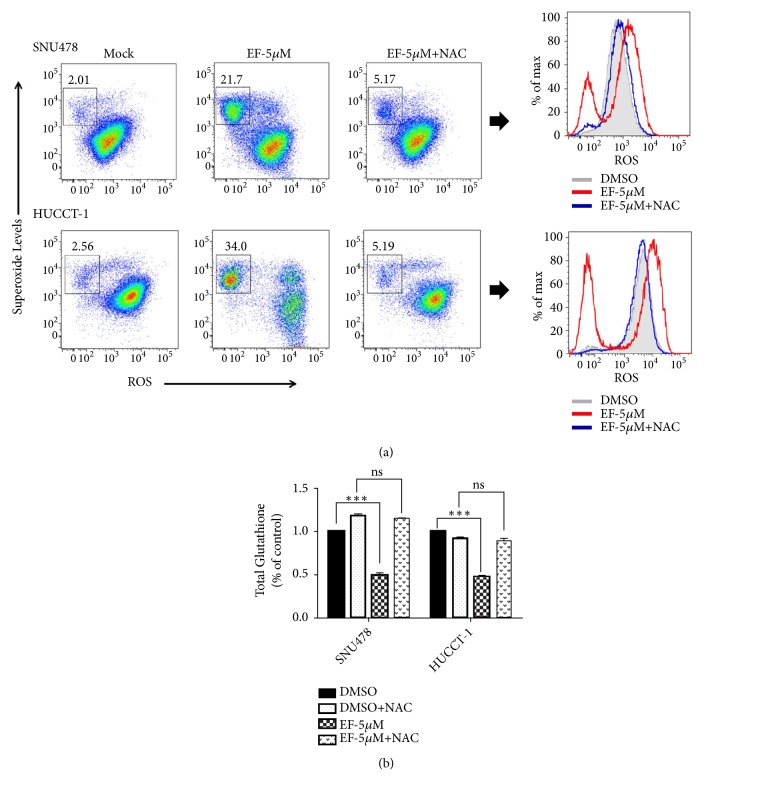
EF24 treatment leads to excessive intracellular ROS production and depletion of total glutathione levels in SNU478 and HuCC-T1 CCC cells. (a) EF24 significantly increased ROS as well as superoxide levels as shown using flow cytometry. This effect, however, was reverted in the presence of the antioxidant N-acetyl cysteine (NAC). (b) EF24 also depleted global glutathione (GSH) levels in both SNU478 and HuCC-T1 cells, whereas in the presence of NAC, GSH levels remained unaffected and were found to be comparable to those found in mock-treated controls using a colorimetric assay (“ns” indicates p>0.05; “*∗∗∗*” indicates p<0.001).

It has previously been shown that many anticancer agents induce ROS-mediated killing of cancer cells by significant accumulation of ROS leading to oxidative damage and cell death [17]. To investigate whether EF24 increases ROS levels in SNU478 and HuCC-T1 cells, we measured total ROS and superoxide levels in EF24-treated cells using flow cytometry. Interestingly, for both cholangiocellular carcinoma cell lines tested, flow cytometry revealed a significant increase in intracellular superoxide free radical (O_2_^∙-^) levels upon treatment with EF24 at 5 *μ*M as compared to mock-treated controls; an increase in intracellular total ROS levels was also observed. As expected, prior treatment with the broad-spectrum antioxidant N-acetyl cysteine (NAC; 5 mM) efficiently attenuated generation of ROS and superoxide in these cells (Figure 3(a)). Additionally, the morphology of the cells was also found to be completely protected from the effects of EF24 when coadministered with NAC (data not shown). However, given the fact that the degree of oxidative stress reflects the balance between the rate of ROS production and the activity of the scavenging systems that detoxify them, we next investigated the effect of EF24 treatment on the status of intracellular glutathione (GSH). As shown in Figure 3(b), treatment with EF24 significantly depleted total GSH levels. Moreover, pretreatment with NAC, a well-described precursor of GSH, was sufficient to completely prevent EF24-mediated GSH depletion.

### 3.4. EF24-Induced Apoptosis Is Rescued by NAC

**Figure 4:**
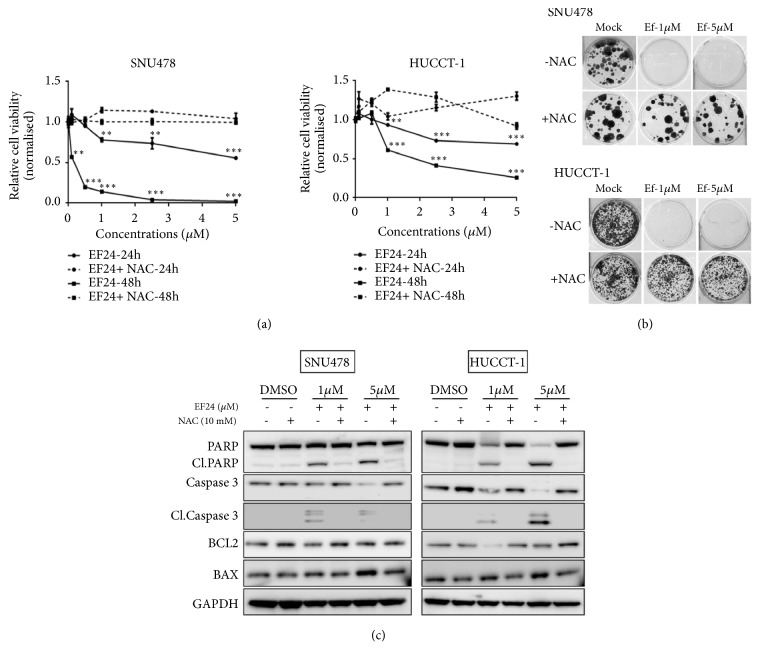
N-acetyl cysteine (NAC) leads to complete abrogation of EF24 antiproliferative and apoptotic activity in cholangiocarcinoma cells. Cell viability (a) and clonogenicity (b) assays performed in the presence of NAC demonstrated complete protection of SNU478 and HuCC-T1 cells from EF24-mediated cytotoxicity. (c) Immunoblot analysis of EF24-treated cells in the presence of NAC also revealed complete blockade of EF24-induced apoptosis in both SNU478 and HuCC-T1 cells (“*∗*” indicates p<0.05; “*∗∗*” indicates p<0.01).

Next, in order to further dissect whether EF24-mediated cytotoxicity and apoptosis in cholangiocarcinoma cell lines is mediated by excessive ROS accumulation, we performed in vitro cell viability, clonogenicity, and apoptosis assays in the absence or presence of NAC (Figure 4). Of note, addition of NAC rescued growth retardation in HuCC-T1 as well as SNU478 cells conferred by EF24 in a dose- and time-dependent manner (Figure 4(a)). While treatment with EF24 administered at doses of 1 or 5 *μ*M, respectively, led to near-complete abrogation of colony formation of SNU478 or HuCC-T1 cells in replating assays, this phenotype, too, was rescued by coadministration of NAC (Figure 4(b)). Finally, Western blot analyses revealed that in both cell lines NAC-mediated rescue of in vitro clonogenicity and net cell growth were accompanied by resuppression of apoptosis biomarkers, cleaved PARP and cleaved caspase 3 (Figure 4(c)).

Thus, taken together, all of these experiments showed that pretreatment with the antioxidant NAC completely prevented EF24-mediated growth inhibition and induction of apoptosis in CCC cells. Our results therefore strongly indicate that EF24 increases oxidative stress, possibly by generating exorbitant levels of ROS but also by undermining the ability of cells to detoxify these oxidants, with this shift in oxidative species balance eventually causing induction of apoptosis and cancer cell death.

### 3.5. EF24 Disrupts Mitochondrial Membrane Potential in Cholangiocarcinoma Cells

**Figure 5:**
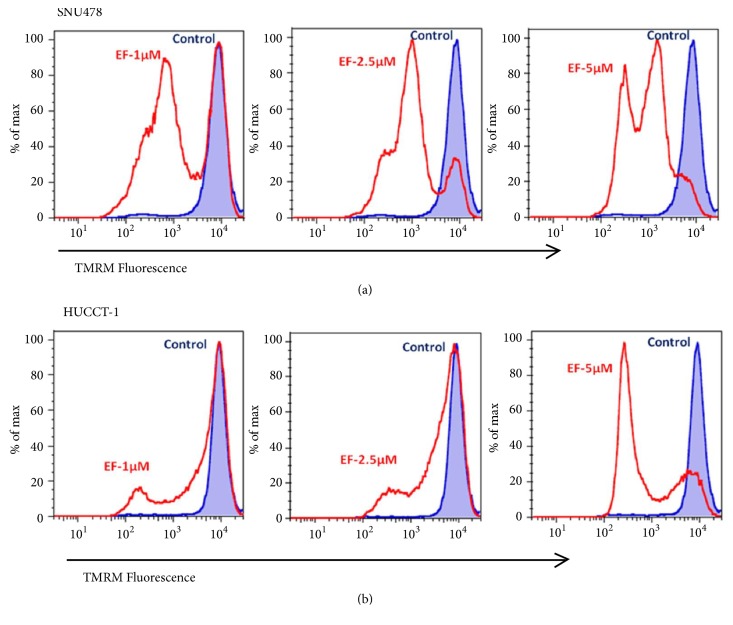
*EF24 decreases the mitochondrial membrane potential of cholangiocarcinoma cells.* Changes in mitochondrial membrane potential were assessed by TMRM staining of SNU478 and HuCC-T1 cells treated with different concentrations of EF24 for 8 hours and the samples were then subjected to flow cytometry analysis.

Dissipation of mitochondrial membrane potential (MMP) is another central mechanism known to be frequently involved in mediating drug-induced apoptosis. Excessive intracellular ROS production has been shown to induce apoptosis by disrupting MMP [28, 29]. Therefore, in order to examine a potential involvement of mitochondria in EF24-induced ROS-mediated apoptosis in CCC cells, we evaluated the induction of mitochondrial membrane damage by EF24. Functional integrity of mitochondrial membranes was assessed by flow cytometry after the cells were labeled with tetramethyl rhodamine methyl ester (TMRM), a dye that accumulates in intact mitochondria and emits red fluorescence. Decrease in fluorescence intensity indicates loss of transmembrane potential [30]. As shown in Figure 5, treatment with EF24 led to depolarization of mitochondria and reduction of membrane potential in a dose-dependent manner, with the maximum drop seen at the highest concentration of EF24 administered (c=5 *μ*M) as indicated by a significant decrease in TMRM fluorescence signal intensity.

### 3.6. EF24 Inhibits Phosphorylation of STAT3

**Figure 6:**
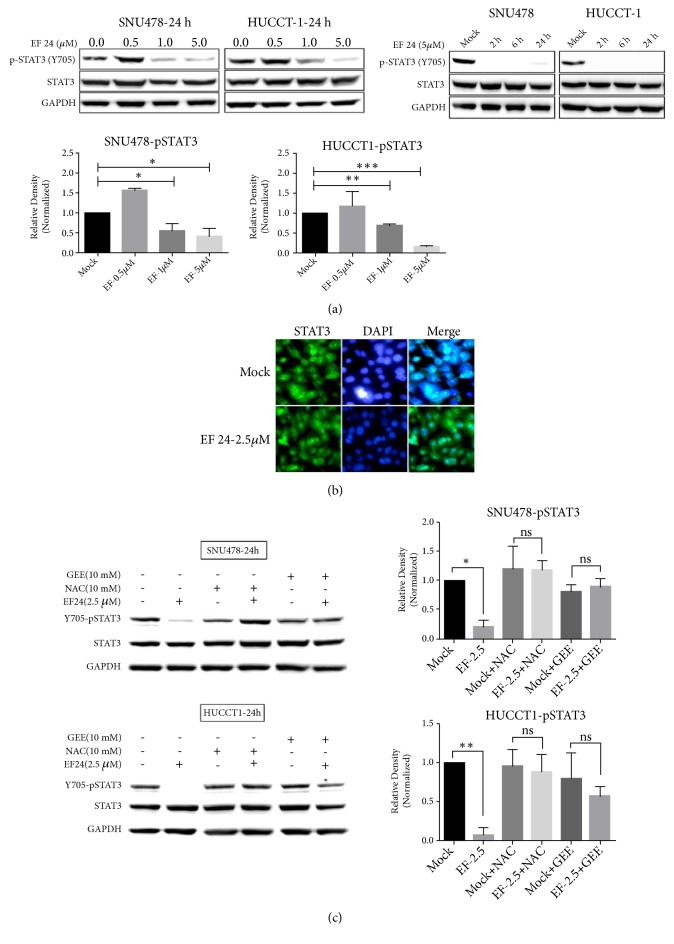
*EF24 inhibits phosphorylation of STAT3.* EF24 decreases Tyr^705^ phosphorylation of STAT3 in a dose- and time-dependent manner in SNU478 and HuCC-T1 cells without affecting total STAT3 expression levels as shown using Western blot analysis (a). Immunofluorescence staining of STAT3 in SNU478 cells confirmed that, in the presence of IL-6, EF24 inactivates STAT3 by inhibiting its phosphorylation and preventing its nuclear translocation (b). Inhibition of STAT3-Tyr^705^ phosphorylation caused by EF24 was reverted by pretreatment with GEE or NAC in Western blot analyses (c) (quantification of Western blot results is shown in the bar diagrams on the right, “*∗*” indicates p<0.05, “*∗∗*” indicates p<0.01, and “*∗∗∗*” indicates p<0.001).

Constitutive STAT3 activation in cholangiocellular carcinomas has previously been shown to be centrally involved in regulating oncogenic gene transcription, tumor progression, and resistance to apoptosis [31–33]. In order to evaluate potential effects of EF24 on STAT3 activation, both cell lines were treated with increasing concentrations of EF24 or solvent for 2, 6, or 24 hours and subjected to Western blot analysis of phosphorylated STAT3 levels. We found that EF24 inhibited STAT3 phosphorylation at tyrosine residue Tyr^705^ in a dose- and time-dependent manner without affecting total STAT3 protein expression levels (Figure 6). Furthermore, immunofluorescence studies were performed to examine the intracellular localization of STAT3 in SNU478 cells in response to EF24 treatment. Fluorescence images revealed that EF24 prevented nuclear translocation of STAT3 even in the presence of IL-6, whereas mock-treated cells showed nuclear accumulation of STAT3 to a larger extent after IL-6 stimulation (Figure 6(b)).

### 3.7. EF24-Induced Glutathione- (GSH-) Depletion Inhibits Phosphorylation of STAT3

In order to dissect the molecular mechanism underlying inhibition of STAT3 phosphorylation by EF24, we hypothesized that increased reactive oxygen species (ROS) levels and rapid drop in intracellular glutathione (GSH) levels might have an important role in this context. Several lines of evidence suggest that STAT3 activation is susceptible to redox regulation where excessive oxidative stress triggers irreversible modification of thiol groups of this protein, finally causing its S-glutathionylation with concomitant inhibition of its phosphorylation [34]. To corroborate whether or not these mechanisms are in fact involved in modulating STAT3 pathway activity in cholangiocarcinoma cells upon administration of EF24, SNU478 and HuCC-T1 cells were pretreated with either 10 mM N-acetyl cysteine (NAC), a precursor to GSH, or a cell permeable glutathione monoethyl ester (GEE; c = 10 mM), which is cleaved to GSH intracellularly, for 2 hours and thereafter with 2.5 *μ*M EF24 for another 6 hours (Figure 6(c)). Western blot analysis revealed that both antioxidants, NAC and GEE, completely reverted inhibition of STAT3 phosphorylation by EF24, hence implying that the protective effects are due to their ability to either increase or sustain the intracellular GSH pool.

### 3.8. EF24 Inhibits Cholangiocarcinoma Growth In Vivo

Exploiting differential redox states between normal and neoplastic cells has long been suggested as potential therapeutic approach to target various cancer tissues [35]. However, the introduction of ROS-generating compounds as potential novel drug candidates has been hampered by several caveats; namely, it was found to be difficult to prove therapeutic selectivity between normal and malignant cells [17]. Therefore, thorough preclinical evaluation of therapeutic efficacy including tests in suitable in vivo model systems is crucial for each such compound in question.

**Figure 7:**
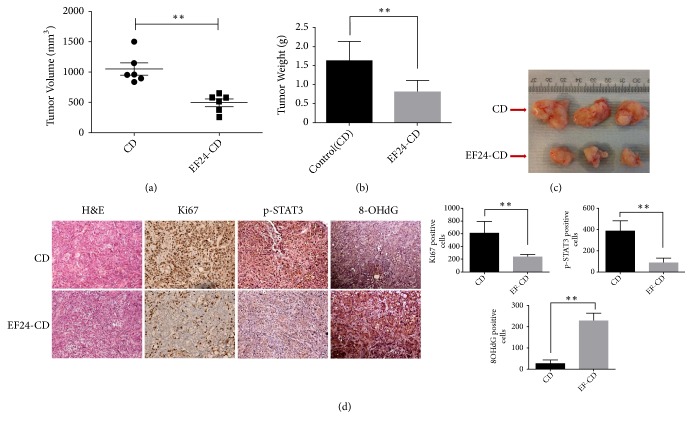
*EF24 inhibits SNU478 xenograft tumor growth in vivo.* SNU478 xenografts treated with EF24-cyclodextrin formulation (EF24-CD) showed significant reduction of mean tumor volumes (a) and tumor weights (b) as compared to cyclodextrin-only (CD) controls. Representative macroscopic photographs of excised tumors harvested at the end of treatment are shown (c). Immunohistochemistry in tissue sections from harvested xenograft tumors confirmed reduced MIB-1 (Ki-67) nuclear staining and significantly reduced levels of pSTAT3 (Tyr^705^) after EF24 treatment (d) (“*∗∗*” indicates p<0.01).

To assess the therapeutic effect of EF24 on tumor growth in vivo, SNU478 xenografts were generated in 6-8-week-old NOD-SCID HPRT mice and allowed to grow to an average size of 80 mm^3^, at which point EF24 solubilized in 10% (w/v) cyclodextrin (EF24-CD) was administered daily to (n=6) mice intraperitoneally for three consecutive weeks. Mice in the control arm (n=6) were mock-treated with cyclodextrin. As shown in Figure 7, treatment with EF24 led to considerable growth retardation of established SNU478 xenograft tumors as compared to controls, with significant differences in average tumor volumes (Figures 7(a) and 7(c)) as well as tumor weights (Figure 7(b)) observed after treatment for three weeks. Thorough necropsy and histological examination of major organs performed at the end of treatment did not reveal any apparent signs of toxicity or difference in mean body weights between the respective treatment arms (data not shown). Of interest, in line with the in vitro data reported above and with the observed in vivo growth inhibition, EF24-treated xenografts showed reduced nuclear Ki-67 staining as compared to mock-treated controls using immunohistochemistry, in line with reduced proliferation (Figure 7(d)). Further, the phosphorylation status of STAT3 found in resected tumor sections correlated with the results obtained in the cell lines and showed markedly reduced levels of p-STAT3 in EF24-treated tumor tissues. Of note, strongly increased expression of 8-OHdG, a biomarker for oxidative damage, was found in EF24-treated tumor tissues, thus confirming EF24-mediated increase in oxidative stress in CCC cells in vivo in the xenograft model utilized here.

## 4. Discussion

In this current study, we show that the curcumin analog EF24 inhibits progression of human cholangiocarcinoma using preclinical in vitro and in vivo model systems and that this compound should thus be further evaluated as potential therapeutic agent for this difficult-to-treat malignancy. These data are in line with a previous report by our own group demonstrating in vivo therapeutic efficacy of a liposomal nanoformulation of EF24 in pancreatic cancer xenografts [36].

Various lines of evidence hint at potential therapeutic efficacy of curcumin and its analog EF24 in a variety of human malignancies [37, 38]. Here we show that EF24 inhibits proliferation, migration, and clonogenicity through induction of apoptosis by increasing oxidative stress in cholangiocarcinoma cells. It has long been suggested that free radicals and increased oxidative stress might contribute to DNA damage and carcinogenesis, and hence antioxidants have been proposed as potential prophylactic agents against neoplasia [39]. However, more recent evidence suggests that this simplistic view does not always seem to be correct and antioxidants have often failed to demonstrate prophylactic properties or have even been found to increase cancer risk, thus indicating more complex underlying regulatory networks modulating oxidative stress in normal as well as in neoplastic cells [40, 41]. It has been noted that oncogenic signals acquired during malignant transformation might both induce ROS generation and hence stimulate cell proliferation through redox-sensitive transcription factors and at the same time promote antioxidant adaptive mechanisms to minimize oxidative damage [17]. In cancer cells, increased overall ROS stress has been found to correlate with tumor aggressiveness and adverse prognosis [42, 43]. One of the mechanisms acquired by cancer cells in order to counteract potential toxic effects conferred by elevated ROS levels appears to be concomitant upregulation of antioxidants [17], as suggested by the observation of increased peroxiredoxin-3 and thioredoxin peroxidase levels in H-ras transformed cells as compared to nontumorigenic counterparts [44]. Moreover, Ras-transformed cells have been shown to be more vulnerable to glutathione depletion with subsequent ROS accumulation and ROS-induced cell death, and ROS-induction by treatment with beta-phenylethyl isothiocyanate (PEITC) led to selective killing of cancer cells and enhanced survival in murine Ras-transformed ovarian cancer xenografts [16]. Therefore, Trachootham et al. hypothesized that, in order to therapeutically target redox-dysregulation in cancer cells that have adapted to oxidative stress by increasing their antioxidant capacity, for example, by upregulation of GSH or GPX, it might not be sufficient to pharmacologically further increase ROS-generation but that a more promising strategy might be to deploy compounds that instead abrogate these adaptive mechanisms, which would then in turn cause a massive accumulation of ROS due to the high basal ROS output in these cells [17]. Our experimental data presented in this current study indicate that EF24 increases oxidative stress in human CCC cell by both increasing ROS levels and at the same time depleting cells of GSH, thus ultimately leading to induction of apoptosis. Of interest and perhaps more importantly, the notion that alteration of redox status might be crucially involved in mediating EF24-induced growth inhibition and apoptosis is further supported by the observation and these effects could be rescued by pretreatment with the antioxidant NAC. Previous studies reported that EF24 and its parent compound curcumin modulate STAT3 phosphorylation [45]. In line with these reports, we were able to show that treatment with EF24 led to reduced phosphorylation of STAT3 at Y705 in cholangiocarcinoma cells. To the best of our knowledge, it is another novel finding of our study that this dephosphorylation might at least in part also be mediated through redox-dependent mechanisms, since this was also partially rescued by antioxidative agents GEE or NAC. Our rescue experiments with NAC and GEE, respectively, provide a strong rationale to suggest that STAT3 phosphorylation might in fact be caused by increased overall oxidative stress. Nevertheless, it can also not completely rule out the fact that STAT3 might also directly contribute to elevated ROS generation, or it might represent mainly a bystander effect.

Since it has been difficult in the past to prove specificity of ROS-mediated elimination of cancer cells [16], it is an important finding of this present study that the observed effects were not limited to in vitro model systems which have repeatedly been shown to be prone to artifacts but were also found using murine xenografts as suitable in vivo models. Of note, we were not only able to show that EF24 treatment caused significantly delayed CCC xenograft growth but also, perhaps equally important, did not observe any obvious signs of toxicity conferred by this compound at the administered doses, as opposed to earlier experience with other compounds targeting redox balance in the past [46, 47]. This is particularly promising with regard to potential further development towards future clinical application in humans.

Our data is in line with previous reports by others suggesting that various phytochemicals exhibit anticancer properties by altering ROS levels [48–50]. In a recent report, Subramani et al. identified the terpenoid lactone Nimbolide as potential therapeutic agent against pancreatic cancer and were able to show that its therapeutic efficacy is mediated at least in part by excessive generation of ROS, supporting the hypothesis that modulating redox potential plays an important role in cancer cell homeostasis and might be successfully exploited as potential therapeutic target. As opposed to our data presented here, Nimbolide seems to mainly act by increasing total ROS levels, while EF24 was found to also curb antioxidative response mechanisms.

Classic cytotoxic agents such as paclitaxel, doxorubicin, or cisplatin have been shown to induce intracellular ROS production, and it was suggested that resistance of cancer cells to these drugs might correlate with and be potentially due to increase in adaptive antioxidant capacity [51]. It is therefore tempting to speculate whether combination regimens composed of such cytostatic drugs plus agents such as EF24 studied here that reduce antioxidative response might carry the potential to overcome resistance and enhance therapeutic selectivity against cancer cells. Evaluation and thorough preclinical characterization of such combinatorial regimens are therefore an interesting approach that should be addressed in future studies. Of note, clinical application of EF24 and its parent compound curcumin has long been hampered by poor bioavailability, and a cyclodextrin-based delivery method such as the one employed here is not likely to be a viable option for administration in humans. However, nanoparticle-based drug delivery platforms have recently been described by our own group as well as by others with the potential to overcome this obstacle [23, 36, 52, 53], so that at least from a technical point of view such combination studies can rightfully be envisioned.

Moreover, more recent studies provide strong evidence to suggest that, in KRAS mutant tumor cells such as pancreatic ductal adenocarcinoma therapeutic synergism of targeting the glutathione antioxidant pathway is to be expected upon combination with inhibitors of the EGFR or AKT pathways [41], and therefore based on our data presented here future combination studies of EF24 with cetuximab of small molecule AKT inhibitors appear to be equally exciting.

## 5. Conclusions

Taken together, the experimental data presented here suggest that the curcumin analog EF24 induces oxidative stress and should be further evaluated as potential therapeutic compound against cholangiocarcinoma.

## Data Availability

The data used to support the findings of this study are included within the article.

## Abbreviations

CCC: Cholangiocellular carcinoma
DMSO: Dimethyl sulfoxide
EDTA: Ethylenediaminetetraacetic acid
GEE: Glutathione monoethyl ester
GSH: Glutathione
IL-6: Interleukin-6
MTS: [3-(4,5-Dimethylthiazol-2-yl)-5-(3-carboxymethoxyphenyl)-2-(4-sulfophenyl)-2H-tetrazolium, inner salt
NAC: N-acetyl cysteine
PBS: Phosphate-buffered saline
PVDF: Polyvinylidene difluoride
ROS: Reactive oxygen species
SSA: Sulfosalicylic acid
TBST: Tris-buffered saline with Tween 20
TMRM: Tetramethyl rhodamine methyl ester.

## Supplementary Materials

Supplementary Materials Supplementary material contains revised figure showing comparative analysis of therapeutic efficacy of EF24 with its parent compound curcumin (*Supplementary Figure 1*).
