# Supplementary figures and images for: EF24 Suppresses Cholangiocellular Carcinoma Progression, Inhibits STAT3 Phosphorylation, and Induces Apoptosis via ROS-Mediated Oxidative Stress
Source: J Oncol. 2019 Mar 4;2019:8701824. doi: 10.1155/2019/8701824 (PMC6425401; doi:10.1155/2019/8701824)

## Slide 1
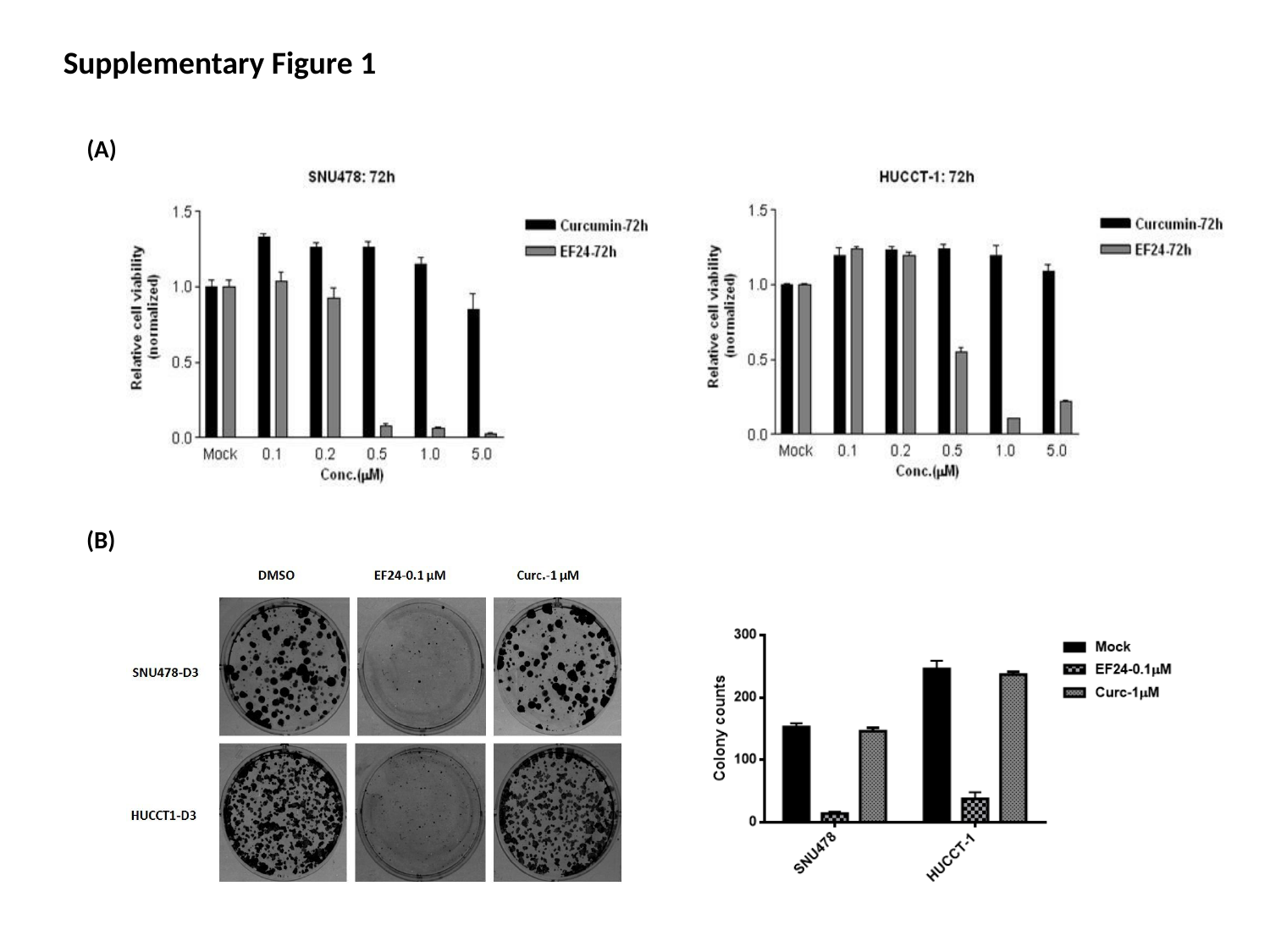

Supplementary Figure 1
(A)
(B)

Supplement: Supplementary Materials — Supplementary material contains revised figure showing comparative analysis of therapeutic efficacy of EF24 with its parent compound curcumin (Supplementary Figure 1). [file 8701824.f1.pptx]
